# A Good Cheap Paste

**Published:** 1877-10

**Authors:** 


### SELECTIONS.

### A GOOD, CHEAP PASTE.

A good, cheap paste that will not foment in warm weather, and which contains no poisonous substances. It makes a paste similar to that used on postage stamps and gummed labels, and is vastly superior to tragacanth, acacia or flour paste, for ordinary use:

| | |
|---|---|
| Dextrine | 2 ounces. |
| Acetic acid | of each 4 drachms. |
| Alcohol | of each 4 drachms. |
| Water | 2½ ounces. |

Mix the dextrine, acetic acid, and water, stirring until thoroughly mixed; then add the alcohol.

